# Promotions of Veterinarians in the Bureau of Animal Industry from December 1, 1900, to July 31, 1901

**Published:** 1901-09

**Authors:** 


### PROMOTIONS OF VETERINARIANS IN THE BUREAU OF ANIMAL INDUSTRY FROM DECEMBER 1, 1900, TO JULY 31, 1901.

THE Hon. James Wilson, from the great agricultural State of Iowa, Secretary of Agriculture in President McKinley's Cabinet, has been one of the foremost of the great men of the country to understand the value, economy, and necessity of having an expert and educated veterinary service for our vast animal interests, and has appreciated that the method to obtain a satisfactory and efficient service is to effect a systematic organization and grade the responsibility of the employés, and then to reward their work and ability, when proven, by promotion and increase of pay.

Secretary Wilson has perfected in the Bureau of Animal Industry under his department a complete organization and fixed competent veterinary service equal to that of any other government. He is able to place, in European ports, in experimental stations, in stockyards, or wherever official veterinary services are needed, expert, educated veterinarians, selected for their known adaptability and learning for the class of work required. While these veterinarians are working directly under other and often outside unofficial people, the Secretary of Agriculture has a record of their professional work, and its quality is known to him through his own inspectors.

To secure properly educated and expert veterinarians the Department of Agriculture pays its

Chief veterinarian $4000 a year; $500 more than a colonel's pay in the United States Army.

Five assistant veterinarians, $2500 a year; a major's pay.

A number $2250 and $2000 a year; a captain's pay or more.

The mass are graded at $1800, $1600, $1400, to which they are advanced shortly after commencing service at $1200 a year.

The system improved by Secretary Wilson is simply a perfection of that previously existing in all other foreign countries in regulating the best and most economical care in their agricultural industries and in their armies.

The United States Army alone, of all great government departments in the world, still retains the obsolete system of employing individual veterinarians who have no head, no organization, no system of supply, and no means of making professional reports; whose responsibility is to a lay officer who may or may not know anything of veterinary matters, and whose work is inspected by the same.

No foreign army has found any friction or trouble in giving to its veterinarians a proper and complete organization and system to work under, and in giving the individuals proper rank and pay.

We trust that our own government will not delay much longer in giving the United States Army a similar service.

The following are the promotions from December 1, 1900, to July 31, 1901:

Dr. M. O. Anderson, South St. Paul, Minn., from $1200 to $1400, and placed in charge of station at Austin, Minn.

Dr. Don C. Ayer, in charge South Omaha, Neb., from $1600 to $1800.

Dr. Levi P. Beechy, South Omaha, Neb., from $1200 to $1400.

Dr. R. J. Blanche, Kansas City, Kan., from $1200 to $1400, and transferred from South St. Joseph, Mo.

Dr. George A. Bond, South St. Joseph, Mo., from $1200 to $1400.

Dr. James J. Brougham, in charge National Stockyards, Ill., from $1600 to $1800.

Dr. John S. Buckley, Washington, D. C., from $1200 to $1400.

Dr. Herman Busman, Chicago, Ill., from $1200 to $1400.

Dr. George W. Butler, placed in charge Eau Claire, Wis.

Dr. Charles H. Canfield, Kansas City, Kan., from $1200 to $1400.

Dr. Thomas W. Carnachan, Kansas City, Kan., from $1200 to $1400.

Dr. Herbert B. Chaney, Kansas City, Kan., from $1200 to $1400.

Dr. Dean G. Cooper, South Omaha, Neb., from $1200 to $1400.

Dr. Robert Darling, Indianapolis, Ind., from $1200 to $1400, and placed in charge San Diego, Cal.

Dr. Frederick T. Dolan, Knoxville, Tenn., from $1200 to $1400.

Dr. Frank C. Eells, Salt Lake City, from $1200 to $1400.

Dr. John William Fink, Washington, D. C., from $1200 to $1400.

Dr. Harrison H. George, Kansas City, Kan., from $1200 to $1400.

Dr. Robert H. Harrison, Milwaukee, Wis., from $1200 to $1400.

Dr. Samuel G. Hendren, New York, N. Y., from $1200 to $1400.

Dr. James G. Hope, Chicago, Ill., from $1200 to $1400.

Dr. Ulysses G. Houck, Chicago, from $1400 to $1600.

Dr. Walter E. Howe, Denver, Col., from $1200 to $1400.

Dr. Benjamin Howes, Island Pond, Vt., from $1200 to $1400, and transferred to Pittsburg, Pa.

Dr. Julius Huelsen, in charge Jersey City, N. J., from $1400 to $1600.

Dr. J. Otis Jacobs, Salt Lake City, from $1200 to $1400.
Dr. George Jobson, Chicago, Ill., from $1200 to $1400.
Dr. John V. Laddey, New York, N. Y., from $1200 to $1400.
Dr. W. B. Lincoln, Nashville, Tenn., from $1200 to $1400.
Dr. George A. Lytle, Chicago, Ill., from $1200 to $1400.
Dr. Alfred F. Martins, Boston, Mass., from $1200 to $1400.
Dr. Chester Miller, St. Louis, Mo., from $1200 to $1400.
Dr. J. C. Milnes, in charge Wichita, Kan., from $1400 to $1600.
Dr. Patrick H. Mullowney, Boston, Mass., from $1200 to $1400.
Dr. Michael T. Naughton, Chicago, Ill., from $1200 to $1400.
Dr. Albert J. Payne, in charge Cincinnati, Ohio, from $1400 to $1600.
Dr. Thomas L. Rice, Cincinnati, Ohio, from $1200 to $1400.
Dr. Albert E. Rishel, Chicago, Ill., from $1200 to $1400.
Dr. Willard A. Savage, Chicago, Ill., from $1200 to $1400.
Dr. Charles A. Schaufler, in charge Philadelphia, Pa., from $1600 to $1800.
Dr. E. C. Schroeder, Bethesda, Md., from $1800 to $2000.
Dr. William H. Smith, Jr., Chicago, Ill., from $1200 to $1400.
Dr. N. C. Sorensen, in charge Indianapolis, Ind., from $1400. to $1600.
Dr. R. P. Steddom, in charge Knoxville, Tenn., from $1400 to $1600.
Dr. Harry O. Thompson, Boston, Mass., from $1200 to $1400.
Dr. William Thompson, in charge Sioux City, Iowa, from $1400 to $1600.
Dr. Richard W. Tuck, Indianapolis, Ind., from $1200 to $1400.
Dr. Henry J. Washburn, Washington, D. C., from $1200 to $1400.
Dr. John B. Wright, South St. Joseph, from $1200 to $1400.

---

*Professional Education in Spain.* The *Gaceta de Med. Zoöl.* gives the reformed scheme of veterinary education issued by the government whereby the university faculty of zoölogical medicine ranks with that of human medicine as in Germany. The course of instruction extends over five years and is very comprehensive, the first degree entitling to practice is Licentiater of Med. Zoöl., the higher ones being Master and Doctor of Med. Zoöl.

